# The impact of neurocognitive and psychiatric disorders on the risk of idiopathic normal pressure hydrocephalus: A bidirectional Mendelian randomization study

**DOI:** 10.1002/brb3.3532

**Published:** 2024-05-23

**Authors:** Yuze He, Zhihao Wang, Mingrong Zuo, Shuxin Zhang, Wenhao Li, Siliang Chen, Yunbo Yuan, Yuan Yang, Yanhui Liu

**Affiliations:** ^1^ Department of Neurosurgery West China Hospital Sichuan University Chengdu China; ^2^ Department of Pediatric Neurosurgery West China Women's and Children's Hospital, Sichuan University West China Second University Hospital Chengdu China; ^3^ Department of Critical Care Medicine West China Hospital Sichuan University Chengdu China

**Keywords:** idiopathic normal pressure hydrocephalus, Mendelian randomization, neuropsychiatric disorders, neuropsychiatric symptoms

## Abstract

**Background:**

Neurocognitive and psychiatric disorders have been proved that they can comorbid more often with idiopathic normal pressure hydrocephalus (iNPH) than general population. However, the potential causal association between these disorders and iNPH has not been assessed. Thus, our study aims to investigate the causal relationship between them based on a bidirectional Mendelian randomization (MR) analysis.

**Methods:**

Random effects of the inverse variance weighted (IVW) method were conducted to obtain the causal association among the neurocognitive disorders, psychiatric disorders, and iNPH. Genome‐wide association studies (GWAS) of 12 neurocognitive and psychiatric disorders were downloaded via the OpenGWAS database, GWAS Catalog, and Psychiatric Genomics Consortium, whereas GWAS data of iNPH were obtained from the FinnGen consortium round 9 release, with 767 cases and 375,610 controls of European ancestry. We also conducted the sensitivity analysis in these significant causal inferences using weighted median model, Cochrane's *Q* test, MR‐Egger regression, MR Pleiotropy Residual Sum and Outlier detect and the leave‐one‐out analysis.

**Results:**

For most of the neurocognitive and psychiatric disorders, no causal association was established between them and iNPH. We have found that iNPH (odds ratio [OR] = 1.030, 95% confidence interval [CI]: 1.011–1.048, *p* = .001) is associated with increased risk for schizophrenia, which failed in validation of sensitivity analysis. Notably, genetically predicted Parkinson's disease (PD) is associated with increased risk of iNPH (OR = 1.256, 95% CI: 1.045–1.511, *p* = .015).

**Conclusion:**

Our study has revealed the potential causal effect in which PD associated with an increased risk of iNPH. Further study is warranted to investigate the association between PD and iNPH and the potential underlying mechanism.

## Graphical abstract

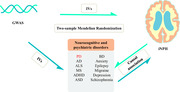

## 1 INTRODUCTION

Idiopathic normal pressure hydrocephalus (iNPH) is an age‐related syndrome characterized by pathological enlargement of the ventricles, accompanied by a normal opening pressure of cerebrospinal fluid (CSF) through lumbar puncture, with the typical characteristics of cognitive impairment, gait disturbance and urinary incontinence (Adams et al., 1965). Initially perceived as a rare clinical disease, iNPH is now increasingly prevalent due to the aggravation of aging, with an average incidence of 179 per 100,000 in individuals over 60 years, as reported in an American study (Alvi et al., 2021; Conn, 2011). Although our understanding of iNPH has improved significantly, the pathophysiologic mechanism remains elusive as it was first reported in 1965 (Hakim & Adams, 1965).

The intricate relationship between iNPH and neurocognitive disorders or psychiatric diseases has not been firmly elucidated, despite numerous studies indicating frequent coexistence with other neurodegenerative disorders such as Alzheimer's disease (AD) and Parkinson's disease (PD) which are acknowledged as comorbidities of iNPH (Kamohara et al., 2020; Ohmichi & Tokuda, 2022). Although the evidence that iNPH is characterized by genetic and pathophysiological mechanisms independence from AD has been reported, the causal association between them remains unclear (Huovinen et al., 2017). Besides, both neuroimaging presentations and shared clinical features like gait disturbance support the notion of PD in iNPH, hindering the differential diagnosis of PD and iNPH (Mostile et al., 2022). These similarities in clinical presentations further complicate the understanding of the relationships between these conditions.

Additionally, iNPH commonly presents concomitantly with psychiatric disorders in elderly individuals, including depression, anxiety, and schizophrenia (SCZ) (Israelsson et al., 2016; Kito et al., 2009; Yoshino et al., 2020). A more frequent possibility of iNPH in older patients with SCZ than in the general population has been reported (Yoshino et al., 2020). Moreover, a prior investigation demonstrated that iNPH patients were more prone to have a positive depression screen and a history of psychiatric disorders (Ghaffari‐Rafi et al., 2020). Collectively, this evidence suggests potential connections between iNPH and both neurocognitive and psychiatric disorders. Moreover, these diseases that coexist in iNPH patients overlap their own clinical characteristics, which makes the differential diagnosis and accurate treatment of iNPH much difficult (Lim et al., 2014). Given that each condition is traditionally treatment independently, there is a crucial need for individualized management plans for iNPH patients following thorough clinical assessments to enable accurate differentiation. However, the direct causal association between neurocognitive and psychiatric disorders and iNPH remains inadequately established. Therefore, further exploration of the relationships between these diseases and iNPH is imperative to deepen our understanding of the disease and enhance accurate diagnosis, thereby enabling the provision of more rationale treatment options for patients.

Mendelian randomization (MR) represents a novel causal inference approach based on genetic variation, addressing certain limitations inherent in conventional randomized controlled trials (Swanson et al., 2017). In MR analysis, based on the data of genome‐wide association studies (GWAS), single nucleotide polymorphisms (SNPs) associated with exposure are leveraged as instrumental variables (IVs) to assess causality between exposure and outcome. It is crucial to note that, although neurocognitive and psychiatric disorders may coexist with iNPH in some cases, the direct causal relationships between them remain unclear (Lim et al., 2014; Yoshino et al., 2020).

In the current investigation, we performed a bidirectional two‐sample MR analysis based on large‐scale GWAS summary statistics of neurocognitive disorders, psychiatric disorders, and iNPH, aiming to identify the causal association between these disorders and iNPH. Our findings furnish bidirectional evidence of causal associations between them, which could help us to elucidate the potential pathogenesis of this age‐disease, making differential diagnosis easier and provide effective intervention strategies possible in the early phases.

## 2 METHODS

### 2.1 Study design and data sources

**FIGURE 1:**
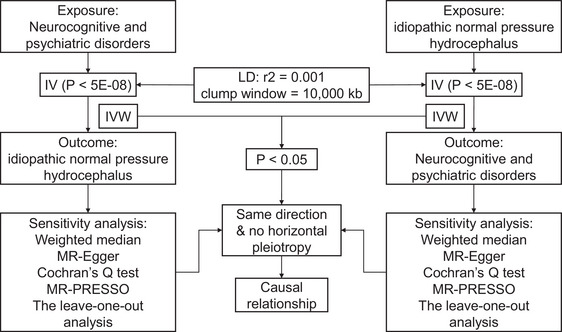
Study design of present research. IVs, instrumental variables; IVW, inverse variance weighted; LD, linkage disequilibrium; MR‐PRESSO, Mendelian Randomization Pleiotropy Residual Sum and Outlier.

In general, the flowchart of this two‐sample MR research is shown in Figure 1. The basic assumptions of MR were satisfied. The IVs must be (i) associated with exposure; (ii) independent of confounders; (iii) associated with outcome only by the exposure (Labrecque & Swanson, 2018). GWAS derived from another 12 of neuropsychiatric disorders were hired, including AD (Kunkle et al., 2019), attention deficit/hyperactivity disorder (Demontis et al., 2019), amyotrophic lateral sclerosis (van Rheenen et al., 2021), anxiety (Otowa et al., 2016), autism spectrum disorder (Grove et al., 2019), bipolar disorder (Hou et al., 2016), epilepsy (International League Against Epilepsy Consortium on Complex Epilepsies, 2018), depression (Howard et al., 2019), migraine (Choquet et al., 2021), multiple sclerosis (International Multiple Sclerosis Genetics Consortium, 2019), PD (Nalls et al., 2019), and SCZ (Howard et al., 2019), with most of the individuals included being European. Complete information of data source is placed in Table 1. As for iNPH, the GWAS data of iNPH were obtained from the FinnGen consortium round 9 release, with 767 cases and 375,610 controls (Kurki et al., 2023). Individuals with a hospitalization history diagnosed as G91.2 in the 10th edition of the International Classification of Diseases were identified as iNPH.

**TABLE 1:** Complete information of data source.

Trait		Abbreviation	Sample size	Diagnostic criteria	Population	Data source	PMID
Idiopathic normal pressure hydrocephalus		iNPH	376,377 (767 cases and 375,610 controls)	G91.2 in ICD‐10		Finngen R9 (https://risteys.finregistry.fi/endpoints/G6_HCNP)	36653562
Neurocognitive disorder	Alzheimer's disease	AD	63,926 (21,982 cases and 41,944 controls)	NINCDSADRDA/DSM IV–V criteria		mrcieu OPEN GWAS database (gwas id: ieu‐b‐2)	30820047
	Amyotrophic lateral sclerosis	ALS	138,086 (27,205 cases and 110,881 controls)	The revised El Escorial criteria		GWAS Catalog (gwas id: GCST90027164)	34873335
	Epilepsy	–	44,889 (15,212 cases and 29,677 controls)	G40.901 in ICD‐10		mrcieu OPEN GWAS database (gwas id: ieu‐b‐8)	30531953
	Migraine	–	58,379	The IHS criteria	European	GWAS Catalog (gwas id: GCST90000016)	34294844
	Multiple sclerosis	MS	115,803 (47,429 cases and 68,374 controls)	2017 McDonald MS criteria		mrcieu OPEN GWAS database (gwas id: ieu‐b‐18)	31604244
	Parkinson's disease	PD	482,730 (33,674 cases and 449,056 controls)	The MDS criteria		mrcieu OPEN GWAS database (gwas id: ieu‐b‐7)	31701892
Psychiatric disorder	Attention deficit/hyperactivity disorder	ADHD	55,374 (20,183 cases (1084, non‐European cases) and 35,191 controls (997, non‐European cases))	F90.0 in ICD‐10	European, North American and Chinese	mrcieu OPEN GWAS database (gwas id: ieu‐a‐1183)	30478444
	Anxiety	–	17,310	DSM‐IV criteria	European	Psychiatric Genomics Consortium (http://www.med.unc.edu/pgc/downloads)	26754954
	Autism spectrum disorder	ASD	46,350 (18,381 cases and 27,969 controls)	F84 in ICD‐10	European	mrcieu OPEN GWAS database (gwas id: ieu‐a‐1185)	30804558
	Bipolar disorder	BD	34,950 (7,647 cases and 27,303 controls)	DSM‐III or DSM‐IV	European	GWAS Catalog (gwas id: GCST003724)	27329760
	Major depression	MD	500,199 (170,756 cases and 329,443 controls)	DSM‐IV criteria	European	mrcieu OPEN GWAS database (gwas id: ieu‐b‐102)	30718901
	Schizophrenia	SCZ	127,906 (52,017 cases and 75,889 controls)	F20 in ICD‐10	European	mrcieu OPEN GWAS database (gwas id: ieu‐b‐5102)	35396580

Abbreviations: AD, Alzheimer's disease; ADHD, attention deficit/hyperactivity disorder; ALS, amyotrophic lateral sclerosis; ASD, autism spectrum disorder; BD, bipolar disorder; DSM, the diagnostic and statistical manual of mental disorders.; GWAS, genome‐wide association studies; ICD, International Classification of Disease; IHS, international headache society; iNPH, idiopathic normal pressure hydrocephalus; MD, major depression; MDS, movement disorders society; MS, multiple sclerosis; NINCDSADRDA, national institute of neurological disorders and stroke‐Alzheimer disease and related disorders; PD, Parkinson's disease; SCZ, schizophrenia.

### 2.2 IVs selection

Normally, SNPs associated with exposures at the genome‐wide threshold (*p* < 5E − 08), and a less strict threshold of *p* < 5E − 06 was adopted if less than four IVs were available. After that, independent SNPs were screened based on European ancestry 1000 Genomes linkage disequilibrium (LD) reference panel. Within a clump window of 10,000 kb, SNP with *r* ^2^ > .001 was considered LD and removed. To select strong IVs, SNPs with low *F*‐statistic (<10) were deleted. To satisfy the third basic assumption of MR, SNPs not reported in the outcome GWAS or directly associated with outcome (*p* < 5E − 08) were also filtered. Rare SNPs with an effect allele frequency less than 0.01 were deleted in an attempt to reduce false positive results. To avoid palindromic SNPs, harmonization was conducted, and mismatched SNPs were excluded (Hemani et al., 2018).

### 2.3 MR analysis

To explore the potential causal effect of iNPH with other neurocognitive and psychiatric disorders, the random effect inverse variance weighted (IVW) analysis was applied, which was unlikely to be affected by heterogeneity (Burgess et al., 2013). Sensitivity analyses were carried out to identify the robustness, including Weighted median model, MR‐Egger regression, Cochran's *Q* test, and MR Pleiotropy Residual Sum and Outlier (MR‐PRESSO). Weighted median model could provide consistent results based on majority analytical weights derived from valid IVs (Burgess et al., 2013). MR‐Egger regression allowed the existence of pleiotropy at a cost of limited statistical power (Bowden et al., 2015). Cochran's *Q* statistics was used to appraise the heterogeneity (Huedo‐Medina et al., 2006). The condition *p* < .05 for Cochran's *Q* indicated the heterogeneity of IVs, whereas *p* < .05 for MR‐Egger intercept indicated the horizontal pleiotropy in the analysis (Verbanck et al., 2018). MR‐PRESSO was used to screen and further remove outliners. Inference of causal relationship should be made on condition that the same direction of effect was reported by IVW, weighted median, and MR‐Egger test, and no horizontal pleiotropy was detected. In addition, the leave‐one‐out analysis was conducted to assess the robustness of the significant results.

### 2.4 Statistical methods

All analyses were performed by R‐4.2.3^1^ with R packages “TwoSampleMR” (Hemani et al., 2018) and “MRPRESSO” (Verbanck et al., 2018). A two‐sided *p* < .05 was considered a significant association between exposure and outcome. Results of IVW, weighted median method, and MR‐Egger regression were displayed as the odds ratio (OR) and 95% confidence interval (CI). Forest plots were utilized to visualize the results of IVW methods. Scatter plot and leave‐one‐out plot were portrayed for significant results in the main analysis.

### 2.5 Ethical approval

All data leveraged in present study had been approved by a relevant review board; thus, further ethical approval of our institutional review board was unnecessary (Kunkle et al., 2019; Demontis et al., 2019; van Rheenen et al., 2021; Otowa et al., 2016; Grove et al., 2019; Hou et al., 2016; International League Against Epilepsy Consortium on Complex Epilepsies, 2018; Howard et al., 2019; Choquet et al., 2021; International Multiple Sclerosis Genetics Consortium, 2019; Nalls et al., 2019; Kurki et al., 2023; Trubetskoy et al., 2022).

## 3 RESULTS

### 3.1 IVs information

The complete results of IVs selection are presented in Tables S1–S24. The total number of IVs for neurocognitive and psychiatric disorders in the analysis of iNPH are 502, whereas the IVs for iNPH ranged from 7 to 20 corresponding to different outcomes. The *F*‐statistics were larger than 10 for all IVs, with their association with exposure stronger than that with the outcome.

### 3.2 The causal effect of iNPH on neurocognitive and psychiatric disorders

**FIGURE 2:**
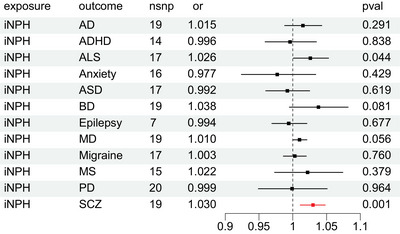
The causal effect of idiopathic normal pressure hydrocephalus on neuropsychiatric disorders.

MR analysis performed with the random effect IVW model is used to assess the causal association of iNPH on these 12 neurocognitive and psychiatric disorders. Complete results are available in Figure 2, and the causal effect of iNPH on SCZ turned out to be a significant result. Specially, iNPH (OR = 1.03, 95% CI: 1.011–1.048, *p* = .001) is associated with an increased risk factor for SCZ. No other significant results present with the causal effect of iNPH on neurocognitive and psychiatric disorders except SCZ. In Table 2 of sensitivity analysis, ADHD and PD demonstrated the opposite effect in weighted median compared with their results of IVW. Besides, ADHD, autism spectrum disorder, migraine, multiple sclerosis, PD, and SCZ also showed opposite effect in MR‐Egger analysis compared with their IVW results. Heterogeneity was detected in the analysis of ADHD (*p* = .017), major depression (*p* = .020), multiple sclerosis (*p* = .014), and PD (*p* = .023). Horizontal pleiotropy of major depression was reported (*p* = .001). Overall, there was no causal relationship of iNPH on these neurocognitive and psychiatric disorders. As to the causal effect of iNPH on SCZ, the causal inference cannot be made due to inconsistent results in sensitivity analysis (Figures S1 and S2).

**TABLE 2:** Sensitivity analysis of normal pressure hydrocephalus (iNPH) on all involved neurocognitive and psychiatric disorders.

Outcome	Weighted median		MR‐Egger regression		Heterogeneity	MR‐PRESSO outlier detect		Pleiotropy
	OR (95% CI)	*p* Value	OR (95% CI)	*p* Value		OR (95% CI)	*p* Value	
AD	1.03 (.99, 1.07)	.145	1.00 (.92, 1.10)	.935	*I* ^2^ = 0%; Cochrane *Q* = 16; *p* = .614	No significant outliers	No significant outliers	Intercept = 0.004; *p* = .803
ADHD	1.00 (.96, 1.04)	.950	1.09 (.98, 1.22)	.150	*I* ^2^ = 50%; Cochrane *Q* = 26; *p* = .017	No significant outliers	No significant outliers	Intercept = −0.031; *p* = .115
ALS	1.03 (1.00, 1.06)	.084	1.00 (.92, 1.09)	.954	*I* ^2^ = 27.2%; Cochrane *Q* = 22; *p* = .144	No significant outliers	No significant outliers	Intercept = 0.010; *p* = .498
Anxiety	.97 (.90, 1.05)	.407	.94 (.77, 1.15)	.583	*I* ^2^ = 0%; Cochrane *Q* = 12; *p* = .679	No significant outliers	No significant outliers	Intercept = 0.011; *p* = .728
ASD	.96 (.92, 1.00)	.052	1.07 (.97, 1.18)	.184	*I* ^2^ = 33.4%; Cochrane *Q* = 24; *p* = .089	No significant outliers	No significant outliers	Intercept = −0.027; *p* = .122
BD	1.02 (.97, 1.08)	.447	1.05 (.92, 1.20)	.458	*I* ^2^ = 0%; Cochrane *Q* = 13; *p* = .817	No significant outliers	No significant outliers	Intercept = −0.005; *p* = .825
Epilepsy	.99 (.96, 1.02)	.425	.93 (.83, 1.04)	.260	*I* ^2^ = 38.8%; Cochrane *Q* = 10; *p* = .133	No significant outliers	No significant outliers	Intercept = 0.021; *p* = .282
MD	1.02 (1.00, 1.03)	.018	1.06 (1.03, 1.09)	.000	*I* ^2^ = 44.4%; Cochrane *Q* = 32; *p* = .020	No significant outliers	No significant outliers	Intercept = −0.017; *p* = .001
Migraine	1.00 (.98, 1.03)	.678	.98 (.92, 1.03)	.437	*I* ^2^ = 0%; Cochrane *Q* = 8; *p* = .948	No significant outliers	No significant outliers	Intercept = 0.009; *p* = .368
MS	1.01 (.95, 1.06)	.771	.90 (.74, 1.10)	.321	*I* ^2^ = 50.1%; Cochrane *Q* = 28; *p* = .014	1.01 (.97, 1.05)	.733	Intercept = 0.039; *p* = .217
PD	1.02 (.96, 1.08)	.578	1.09 (.92, 1.28)	.341	*I* ^2^ = 42.6%; Cochrane *Q* = 33; *p* = .023	No significant outliers	No significant outliers	Intercept = −0.029; *p* = .312
SCZ	1.03 (1.01, 1.06)	.008	.98 (.93, 1.03)	.427	*I* ^2^ = 15.7%; Cochrane *Q* = 21; *p* = .262	No significant outliers	No significant outliers	Intercept = 0.017; *p* = .064

Abbreviations: AD, Alzheimer's disease; ADHD, attention deficit/hyperactivity disorder; ALS, amyotrophic lateral sclerosis; ASD, autism spectrum disorder; BD, bipolar disorder; CI, confidence interval; iNPH, idiopathic normal pressure hydrocephalus; MD, major depression; MR‐PRESSO, Mendelian Randomization Pleiotropy Residual Sum and Outlier; MS, multiple sclerosis; OR, odds ratio; PD, Parkinson's disease; SCZ, schizophrenia.

### 3.3 The causal effect of neurocognitive disorders on iNPH

**FIGURE 3:**
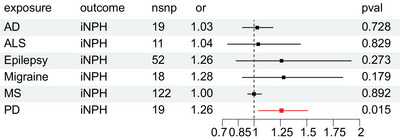
The causal effect of neurocognitive disorders on idiopathic normal pressure hydrocephalus.

On the contrary, we investigated the causal effect of neurocognitive disorders on iNPH. All the results are presented in Figure 3. Notably, PD is the significant positive result with an increased risk association for iNPH. Based on the IVW results, PD is associated with an increased risk factor for iNPH [OR = 1.256, 95% CI: 1.045–1.511, *p* = .015]. The sensitivity analysis results are available in Table 3. No heterogeneity or horizontal pleiotropy was detected. No significant outliers were detected in MR‐PRESSO. The results of weighted median analysis and MR‐Egger analysis were consistent with the IVW results shown in Figure S3. The leave‐one‐out analysis also demonstrated the robust causal effect of the relationship between PD and iNPH in Figure S4, since removing any one IV did not shift the results of PD on iNPH. In this part, we discovered that PD had an increased causal association with iNPH.

**TABLE 3:** Sensitivity analysis of neurocognitive disorders on normal pressure hydrocephalus (iNPH).

Exposure	Weighted median		MR‐Egger regression		Heterogeneity	MR‐PRESSO outlier detect		Pleiotropy
	OR (95% CI)	*p* Value	OR (95% CI)	*p* Value		OR (95% CI)	*p* Value	
AD	1.02 (.83, 1.25)	.867	.99 (.81, 1.20)	.897	*I* ^2^ = 0%; Cochrane *Q* = 17; *p* = .496	No significant outliers	No significant outliers	Intercept = 0.012; *p* = .578
ALS	1.16 (.75, 1.79)	.503	1.62 (.83, 3.17)	.190	*I* ^2^ = 0%; Cochrane *Q* = 7; *p* = .731	No significant outliers	No significant outliers	Intercept = −0.061; *p* = .167
Epilepsy	1.08 (.61, 1.92)	.795	1.28 (.27, 5.97)	.756	*I* ^2^ = 11.2%; Cochrane *Q* = 57; *p* = .249	No significant outliers	No significant outliers	Intercept = −0.001; *p* = .988
Migraine	1.35 (.83, 2.19)	.227	2.16 (.59, 7.95)	.265	*I* ^2^ = 10.8%; Cochrane *Q* = 19; *p* = .325	No significant outliers	No significant outliers	Intercept = −0.044; *p* = .427
MS	1.08 (.97, 1.20)	.171	1.03 (.93, 1.14)	.623	*I* ^2^ = 0.8%; Cochrane *Q* = 122; *p* = .459	No significant outliers	No significant outliers	Intercept = −0.005; *p* = .601
PD	1.14 (.9, 1.46)	.281	1.18 (.64, 2.17)	.598	*I* ^2^ = 0%; Cochrane *Q* = 9; *p* = .961	No significant outliers	No significant outliers	Intercept = 0.010; *p* = .838

Abbreviations: AD, Alzheimer's disease; ALS, amyotrophic lateral sclerosis; CI, confidence interval; iNPH, idiopathic normal pressure hydrocephalus; MR‐PRESSO, Mendelian Randomization Pleiotropy Residual Sum and Outlier; MS, multiple sclerosis; OR, odds ratio; PD, Parkinson's disease.

### 3.4 The causal effect of psychiatric disorders on iNPH

**FIGURE 4:**
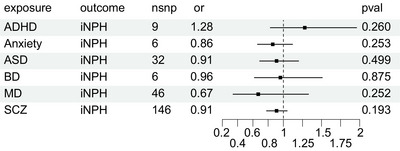
The causal effect of psychiatric disorders on idiopathic normal pressure hydrocephalus.

As to psychiatric disorders, all details of the IVW analysis are shown in Figure 4. The results implicated no suggestive evidence of the causal effect of psychiatric disorders on iNPH. No horizontal pleiotropy existed in the results of sensitivity analysis in Table 4.

**TABLE 4:** Sensitivity analysis of psychiatric disorders on normal pressure hydrocephalus (iNPH).

Exposure	Weighted median		MR‐Egger regression		Heterogeneity	MR‐PRESSO outlier detect		Pleiotropy
	OR (95% CI)	*p* Value	OR (95% CI)	*p* Value		OR (95% CI)	*p* Value	
ADHD	1.41 (.80, 2.48)	.232	1.25 (.22, 7.04)	.806	*I* ^2^ = 0%; Cochrane *Q* = 4; *p* = .883	No significant outliers	No significant outliers	Intercept = 0.002; *p* = .982
Anxiety	.72 (.50, 1.03)	.074	.62 (.28, 1.40)	.313	*I* ^2^ = 10.9%; Cochrane *Q* = 6; *p* = .346	No significant outliers	No significant outliers	Intercept = 0.063; *p* = .455
ASD	.99 (.67, 1.44)	.943	.63 (.27, 1.45)	.287	*I* ^2^ = 6.2%; Cochrane *Q* = 33; *p* = .367	No significant outliers	No significant outliers	Intercept = 0.034; *p* = .368
BD	.88 (.63, 1.23)	.454	.81 (.28, 2.37)	.720	*I* ^2^ = 65%; Cochrane *Q* = 14; *p* = .014	No significant outliers	No significant outliers	Intercept = 0.033; *p* = .738
MD	.72 (.31, 1.68)	.443	2.42 (.04, 146)	.674	*I* ^2^ = 30.6%; Cochrane *Q* = 65; *p* = .028	No significant outliers	No significant outliers	Intercept = −0.039; *p* = .537
SCZ	.90 (.73, 1.12)	.352	1.35 (.75, 2.43)	.320	*I* ^2^ = 2.6%; Cochrane *Q* = 149; *p* = .397	No significant outliers	No significant outliers	Intercept = −0.027; *p* = .174

Abbreviations: ADHD, attention deficit/hyperactivity disorder; ASD, autism spectrum disorder; BD, bipolar disorder; CI, confidence interval; iNPH, idiopathic normal pressure hydrocephalus; MD, major depression; MR‐PRESSO, Mendelian Randomization Pleiotropy Residual Sum and Outlier; OR, odds ratio; SCZ, schizophrenia.

## 4 DISCUSSION

In this study, we conducted a comprehensive assessment to elucidate the causal effect between these 12 neurocognitive and psychiatric disorders and iNPH. The results derived from the MR analysis in our study revealed that PD is associated with an increased risk for iNPH.

Unlike the well‐defined etiology of secondary hydrocephalus known as obstructive hydrocephalus, there is no apparent restriction of CSF outflow in iNPH. Therefore, misdiagnosing iNPH is not new due to the following reasons: (i) inconsistent understanding of iNPH across various regions (Tan et al., 2021), (ii) overlapping symptoms, such as dementia and gait disturbance, with other diseases like AD and PD (Morishita et al., 2010; Sakurai et al., 2022), and (iii) elderly individuals often present with multiple concurrent conditions, obscuring the primary disease (Mori et al., 2012). Given the overlapping characteristics between these disorders and iNPH, meticulous clinical assessments are essential for iNPH to differentiate diagnosis and provide appropriate managements as each disease necessitates distinct treatment. The identification of the causal relationship among neurocognitive disorders, psychiatric diseases, and iNPH based on our MR analysis can enhance the precision of iNPH diagnosis and treatment.

Nowadays, the prevalence iNPH among elderly is elevated, which urged development in its etiology, pathophysiology, and management (Conn, 2011; Alvi et al., 2021). Although the definitive etiology remains unclear, the pathologically enlarged ventricles suggest a potential association with disrupted CSF homeostasis as one contributing factor for iNPH. A widely accepted consensus posits that the enlargement of brain ventricles in iNPH is associated with compromised CSF homeostasis (Wang et al., 2020). Additionally, previous studies have demonstrated that iNPH frequently coexists with these neurocognitive and psychiatric disorders, promoting a further exploration of the potential associations that were not previously elucidated (Yoshino et al., 2020; Israelsson et al., 2016; Kito et al., 2009). The presence of comorbid PD or PD dementia has been shown to exacerbate the clinical course of iNPH, underscoring the need to unravel the potential association between iNPH and PD for more informed treatment planning (Sakurai et al., 2022).

An unobstructed and hyperdynamic CSF circulation pathway in iNPH patient is typically observed (Bradley, 2015). In iNPH, reduced arterial pulpability disturbs CSF balance, intensifying the pulsatility of the aqueduct (Wang et al., 2020). This restricted arterial pulsatility often coincides with heightened pulsatility in the aqueduct and capillaries loss which results in chronic ischemia and hypoperfusion around the ventricles, potentially contributing to increased CSF outflow resistance, leading to ventriculomegaly and reduced intracranial compliance (Oliveira et al., 2019). A study focusing on blood flow patterns identified prevalent hypoperfusion in the ventrolateral frontal cortex, supramarginal gyrus, and temporal cortical regions in iNPH (Kang et al., 2023). Notably, the hypoperfusion of the right frontal lobe is also closely related to urinary incontinence (Sakakibara et al., 2012). However, hyperperfusion in other regions is also observed, warranting further investigation into cerebral blood flow dynamics in iNPH (Kang et al., 2023). Furthermore, the central role of CSF dynamics in various neurocognitive disorders and psychiatric disorders progress, including iNPH and SCZ, has been established (Kartalcı et al., 2021). Abnormal CSF dynamics, as evidenced by ventricular enlargement in magnetic resonance imaging, coexist with psychiatric disorders like SCZ (Kartalcı et al., 2021). Besides, a high occurrence of hydrocephalus has been reported in individuals with autism spectrum disorder despite the unclear role of abnormal CSF dynamics in this disease (Munch et al., 2021). Abnormal CSF dynamics have also been confirmed to mediate amyotrophic lateral sclerosis, sharing similarities with iNPH in increased aqueduct pulsatility (Ng Kee Kwong et al., 2020). Above all, although a definitive causal relationship is yet to be established, we assumed that CSF disorders may represent a shared pathophysiological progress in iNPH and both neurocognitive and psychiatric disorders. Further investigations are essential to comprehensively comprehend the role of abnormal CSF dynamics.

It is well‐established that the impaired cortico‐striatal system, affecting dopamine levels, leads to motor symptoms and cognitive impairment in both PD and iNPH patients (Todisco et al., 2021; Matsumoto, 2015). The enlargement of ventricles resulting from CSF retention can exert pressure adjacent brain tissue, particularly the striatum. A deformed striatum, especially the caudate, contributes to the motor symptoms observed in iNPH, which is a potential reason of the dysfunctional striatum dopaminergic pathway (Sakurai et al., 2022). The decreased binding of striatal dopamine reuptake transporters and D2 receptors in iNPH may be one reason for the motor impairment, as evidenced by the inefficacy of L‐dopa in rescuing iNPH motor symptoms (Pozzi et al., 2021; Ouchi et al., 2007). In addition, this decrease in dopamine levels is consistent with the severity of parkinsonism, indicating that there are some potential mechanistic links between PD and iNPH in the impaired dopamine pathway (Pozzi et al., 2021). However, conflicting study has shown that levodopa therapy can improve gait parameters in iNPH (Onder et al., 2022). These inconsistent results demonstrate that the specific mechanisms of gait disturbance in iNPH and the relationship between iNPH and PD are not fully comprehended. Considering shared characteristics and findings from our MR analysis, we propose a hypothesis that PD may exacerbate iNPH by aggravating the dopaminergic deficit, particularly in the caudate nucleus with a more symmetric presentation in iNPH patients (Pozzi et al., 2021). Besides, different management strategies are warranted due to differences in levodopa response between iNPH and PD, especially considering the frequent coexistence of iNPH and PD (Morishita et al., 2010). A prospective clinical study revealed that shunt surgery could reverse the striatal dysfunction of dopamine reuptake transporters, a response not observed in patients only with PD (Morishita et al., 2010; Todisco et al., 2021). Therefore, further studies should be performed to investigate the causal relationship based on lager clinical dataset and the progression from PD to iNPH so that doctors could accurately diagnose and individualize treatments for each patient.

Cognitive impairment is another shared characteristic between iNPH and neurocognitive disorders or psychiatric disorders. Although different cognitive impairments in the early phase have been reported, the direct relationship between iNPH and cognitive impairment in PD is unclear (Picascia et al., 2019). The same pathophysiology of cognitive impairment observed in iNPH and PD, affecting cortical areas and basal ganglia circuits, partially elucidates the close association of cognitive impairment in these two diseases (Picascia et al., 2019). As to iNPH, the glymphatic system, a crucial delicate regulator of CSF balance, is known to participate in dementia in iNPH individuals (Tan et al., 2021; Ringstad et al., 2017). Studies have shown that restricted arterial pulsatility impairs the glymphatic system, leading to CSF retention and reflux to the ventricles (Bonney et al., 2022). Recognized as an integral component of brain clearance (Reeves et al., 2020), the glymphatic system's reduced flow fails to eliminate metabolic wastes, including accumulated inflammatory cytokines like ILs and TNFα in the cerebral nerve system, which could contribute to dementia. Although no direct causal relationship was identified between iNPH and AD based on our MR study, the decreased clearance of amyloid‐β (Aβ) resulting from the glymphatic system impairment also contributes to cognitive impairment in both iNPH and AD patients (Reeves et al., 2020). Although there is a widely acknowledged association between AD and iNPH, based on the overlapping pathophysiology above, our MR results do not establish a causal relationship between them. Additional investigations are needed to assess the relationship between AD and iNPH. Nevertheless, all these changes collectively result in the dementia in iNPH individuals. More details need to explore for distinguishing the cognitive impairment between iNPH and both neurocognitive and psychiatric disorders and to unravel the pathophysiological progress of cognitive impairment in both PD and iNPH.

Our study has several limitations. First, most the samples involved in the MR analysis were European, which would lack of full representation of other ethnics. Second, the cases of iNPH are not sufficient, requiring the GWAS studies with more samples, stricter criteria and deeper sequencing. Third, there are limitations of MR method, in which other evidence is needed to confirm for our results.

## 5 CONCLUSION

In conclusion, we conducted a bidirectional MR analysis among common neurocognitive disorders, psychiatric disorders, and iNPH. We found that the PD is associated with an increased risk of iNPH, in which the direct relationship is not elucidated before. More work should be done to further confirm it.

## AUTHOR CONTRIBUTIONS

Yuze He, Zhihao Wang, Mingrong Zuo, and Yanhui Liu designed the study, analyzed, and interpreted the data. Yuze He edited the manuscript. Wenhao Li, Siliang Chen, Yunbo Yuan, Yuan Yang, Shuxin Zhang, and Yanhui Liu collected the data and revised the manuscript. All authors drafted and reviewed the final manuscript.

## CONFLICT OF INTEREST STATEMENT

The authors declare no conflicts of interest.

## FUNDING INFORMATION

Sichuan Science and Technology Program, Grant Number: 2023YFQ0002; Sichuan Science and Technology Program, Grant Number: 2023YFG0127; Sichuan Provincial Foundation of Science and Technology, Grant Number: 2023NSFSC1867.

### PEER REVIEW

The peer review history for this article is available at https://publons.com/publon/10.1002/brb3.3532.

## Supporting information

Figure S1 Scatter plot of idiopathic normal pressure hydrocephalus to schizophrenia.

Figure S2 Leave‐one out of idiopathic normal pressure hydrocephalus to schizophrenia.

Figure S3 Scatter plot of Parkinson's disease to idiopathic normal pressure hydrocephalus.

Figure S4 Leave‐one out of Parkinson's disease to idiopathic normal pressure hydrocephalus.

Tables S1–S24 List of all detailed information of instrumental SNPs. Abbreviations: iNPH, idiopathic normal pressure hydrocephalus; AD, Alzheimer's disease; ALS, amyotrophic lateral sclerosis; MS, multiple sclerosis; PD, Parkinson's disease; ADHD, attention deficit/hyperactivity disorder; ASD, autism spectrum disorder; BD, bipolar disorder; MD, major depression; SCZ, schizophrenia; SNP, single nucleotide polymorphism; chr, chromosome; pos, position; EA, effect allele; OA, other allele; se, standard error; eaf, effect allele frequency.

## Data Availability

No original data were generated in our study. Summarized GWAS data for neuropsychiatric disorders were downloaded via the MRC‐IEU OpenGWAS database,^2^ GWAS Catalog,^3^ and Psychiatric Genomics Consortium.^4^ GWAS of iNPH was from FinnGen consortium round 9 release,^5^ seeing Table 1 for complete information (Kurki et al., 2023).

## NOTES

1
https://www.R‐project.org/
