# Supplementary figures and images for: The impact of neurocognitive and psychiatric disorders on the risk of idiopathic normal pressure hydrocephalus: A bidirectional Mendelian randomization study
Source: Brain Behav. 2024 May 23;14(5):e3532. doi: 10.1002/brb3.3532 (PMC11112403; doi:10.1002/brb3.3532)

# MR Test

- Inverse variance weighted
- MR Egger
- Simple mode
- Weighted median
- Weighted mode

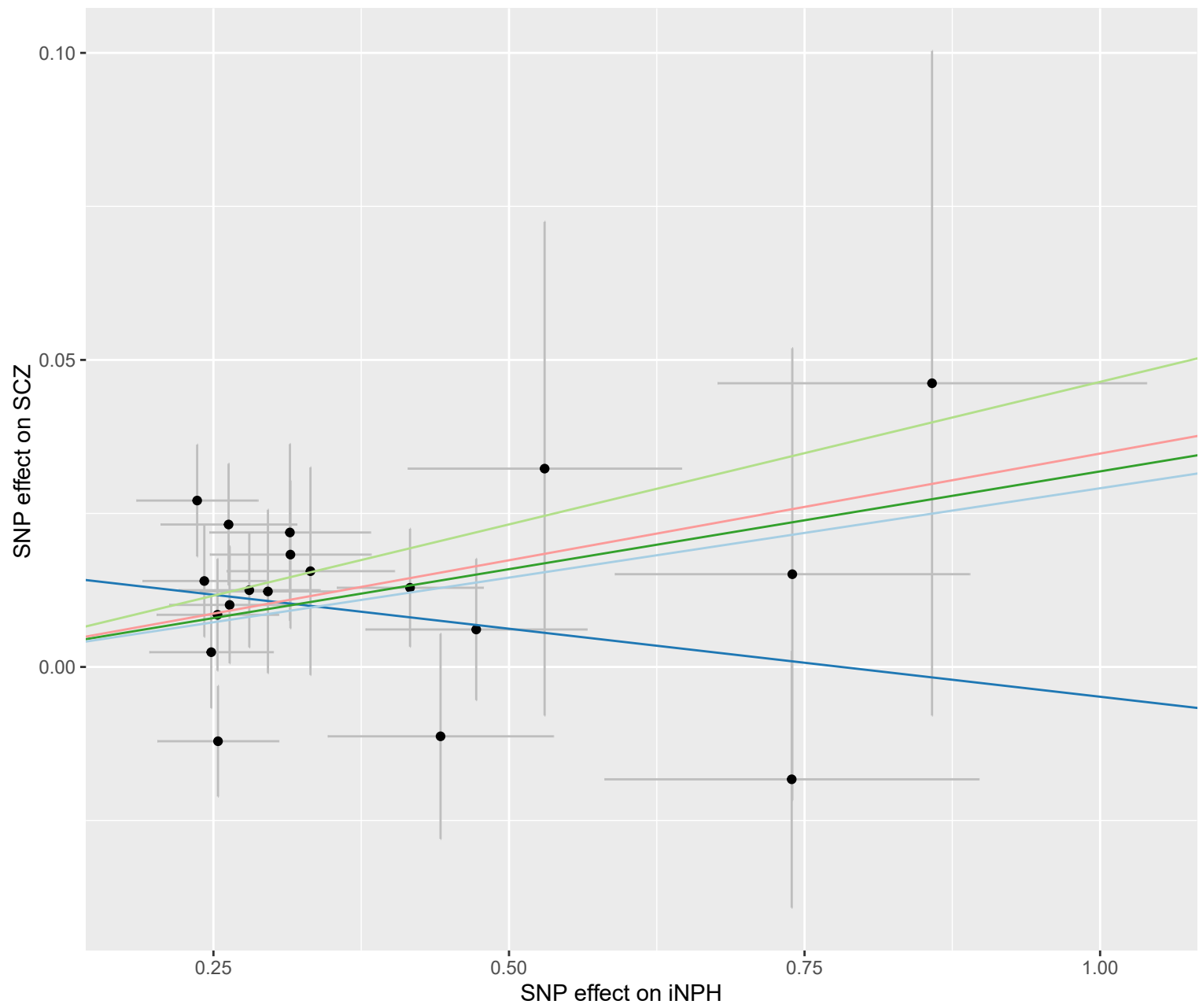

Supplement: Supplementary file 1 — Figure S1 Scatter plot of idiopathic normal pressure hydrocephalus to schizophrenia. [file BRB3-14-e3532-s001.pdf]

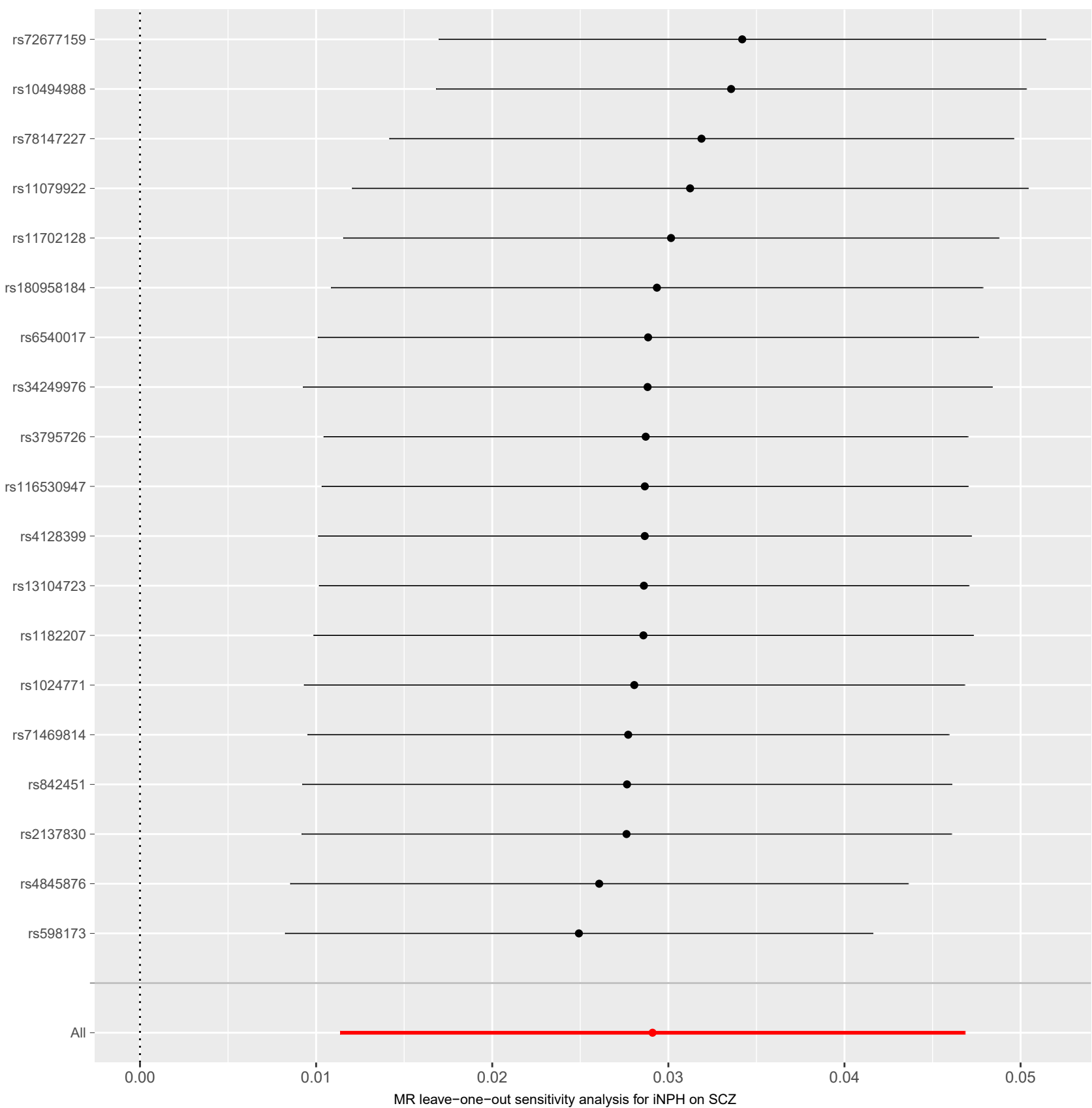

Supplement: Supplementary file 2 — Figure S2 Leave‐one out of idiopathic normal pressure hydrocephalus to schizophrenia. [file BRB3-14-e3532-s005.pdf]

# MR Test

- Inverse variance weighted
- MR Egger
- Simple mode
- Weighted median
- Weighted mode

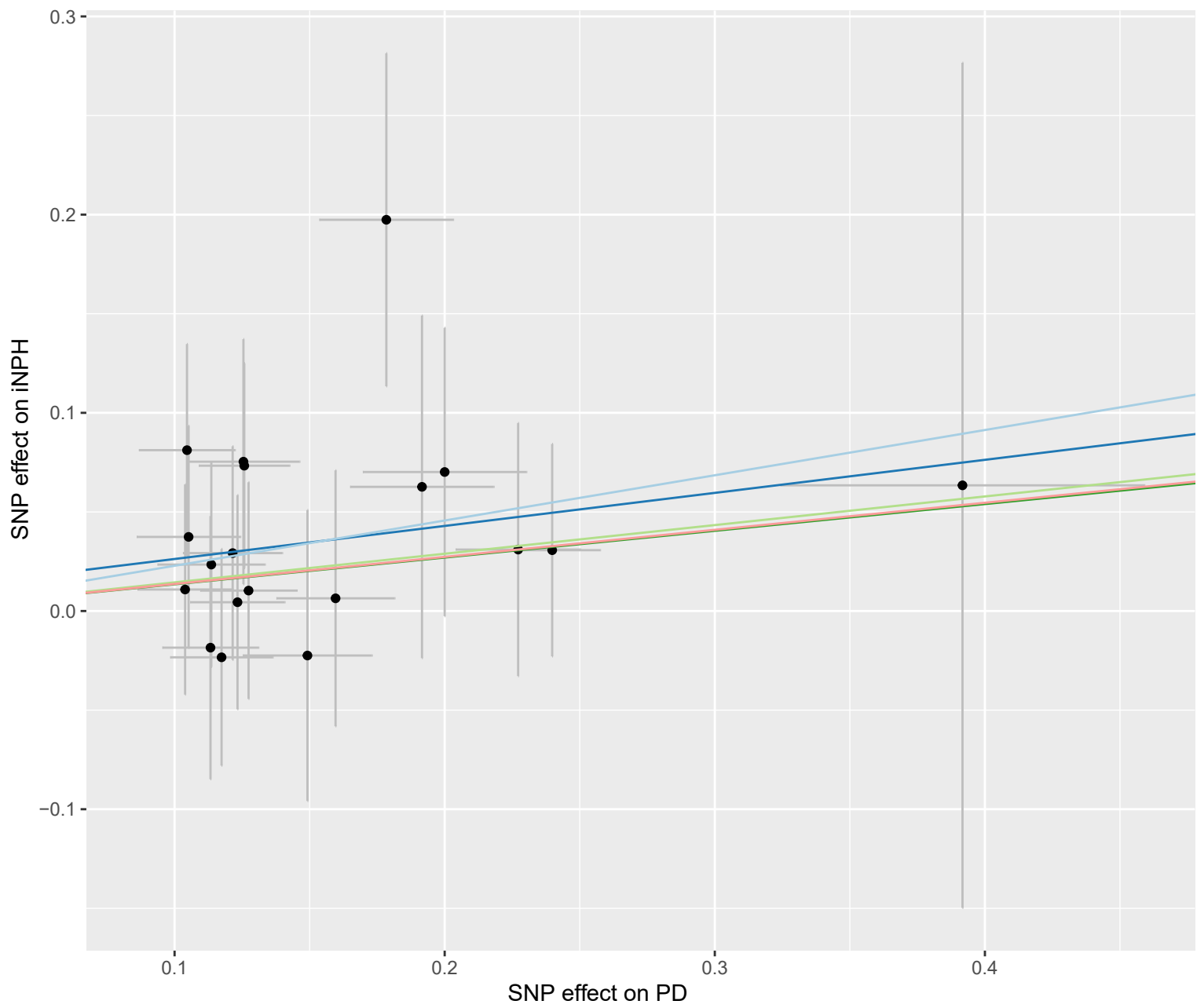

Supplement: Supplementary file 3 — Figure S3 Scatter plot of Parkinson's disease to idiopathic normal pressure hydrocephalus. [file BRB3-14-e3532-s002.pdf]

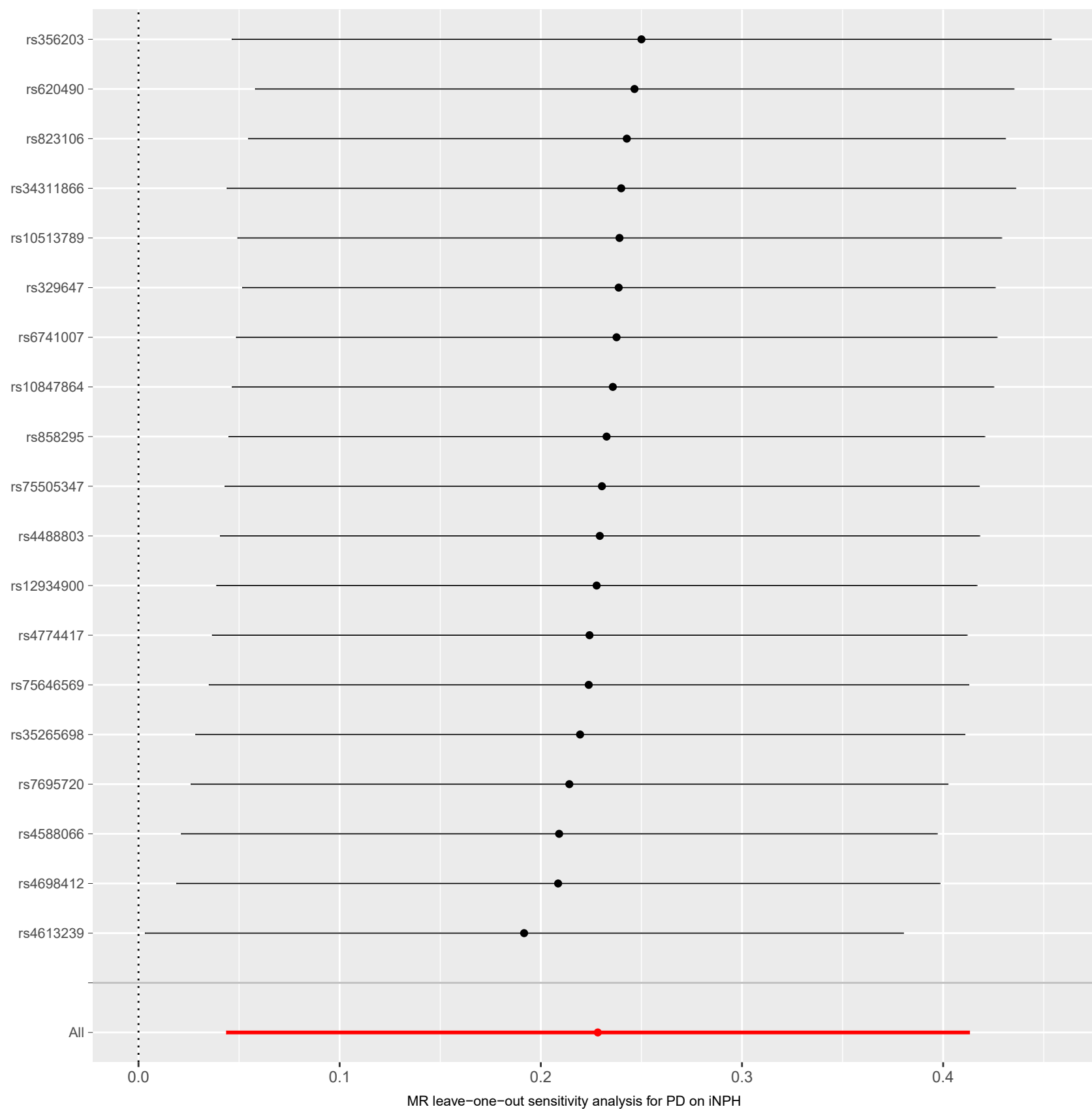

Supplement: Supplementary file 4 — Figure S4 Leave‐one out of Parkinson's disease to idiopathic normal pressure hydrocephalus. [file BRB3-14-e3532-s003.pdf]
